# Plasma L-Cystine/L-Glutamate Imbalance Increases Tumor Necrosis Factor-Alpha from CD14+ Circulating Monocytes in Patients with Advanced Cirrhosis

**DOI:** 10.1371/journal.pone.0023402

**Published:** 2011-08-17

**Authors:** Eiji Kakazu, Yoshiyuki Ueno, Yasuteru Kondo, Jun Inoue, Masashi Ninomiya, Osamu Kimura, Yuta Wakui, Koji Fukushima, Keiichi Tamai, Tooru Shimosegawa

**Affiliations:** 1 Division of Gastroenterology, Tohoku University Graduate School of Medicine, Aobaku, Sendai, Japan; 2 Miyagi Cancer Center, Natori, Japan; Charité-University Medicine Berlin, Germany

## Abstract

**Background and Aims:**

The innate immune cells can not normally respond to the pathogen in patients with decompensated cirrhosis. Previous studies reported that antigen-presenting cells take up L-Cystine (L-Cys) and secrete substantial amounts of L-Glutamate (L-Glu) via the transport system Xc- (4F2hc+xCT), and that this exchange influences the immune responses. The aim of this study is to investigate the influence of the plasma L-Cys/L-Glu imbalance observed in patients with advanced cirrhosis on the function of circulating monocytes.

**Methods:**

We used a serum-free culture medium consistent with the average concentrations of plasma amino acids from patients with advanced cirrhosis (ACM), and examined the function of CD14+ monocytes or THP-1 under ACM that contained 0–300 nmol/mL L-Cys with LPS. In patients with advanced cirrhosis, we actually determined the TNF-alpha and xCT mRNA of monocytes, and evaluated the correlation between the plasma L-Cys/L-Glu ratio and TNF-alpha.

**Results:**

The addition of L-Cys significantly increased the production of TNF alpha from monocytes under ACM. Monocytes with LPS and THP-1 expressed xCT and a high level of extracellular L-Cys enhanced L-Cys/L-Glu antiport, and the intracellular GSH/GSSG ratio was decreased. The L-Cys transport was inhibited by excess L-Glu. In patients with advanced cirrhosis (n = 19), the TNF-alpha and xCT mRNA of monocytes were increased according to the Child-Pugh grade. The TNF-alpha mRNA of monocytes was significantly higher in the high L-Cys/L-Glu ratio group than in the low ratio group, and the plasma TNF-alpha was significantly correlated with the L-Cys/L-Glu ratio.

**Conclusions:**

A plasma L-Cys/L-Glu imbalance, which appears in patients with advanced cirrhosis, increased the TNF-alpha from circulating monocytes via increasing the intracellular oxidative stress. These results may reflect the immune abnormality that appears in patients with decompensated cirrhosis.

## Introduction

Circulating levels of proinflammatory cytokines such as TNF-alpha, IL-1 beta and IL-6 are increased in patients with cirrhosis [1], [2], [3]. Endotoxemia has been assumed to be responsible for the increased of such cytokines in patients with cirrhosis [4], because the activation of monocytes, macrophages and dendritc cells (DCs) by lipopolysaccharide (LPS) plays a key role in the pathogenesis of cytokine overproduction. This overproduction of proinflammatory cytokines leads to various complications, such as spontaneous bacterial peritonitis (SBP) and hepatorenal syndrome (HRS) in patients with advanced cirrhosis [5], [6].

On the other hand, various types of amino acid imbalance appear in the plasma of patients with decompensated cirrhosis, since the liver plays a major role in metabolism involving glucose, lipids, vitamins,minerals and amino acids. An imbalance of plasma amino acids, with decreased levels of branched-chain amino acids (BCAAs) and increased levels of aromatic amino acids (AAAs), is commonly seen in patients with advanced cirrhosis [7]. Previously, we reported that extracellular branched-chain amino acids (BCAAs) regulate the maturation and function of monocyte derived dendritic cells [8], and that the addition of branched chain amino acids enhances the maturation and function of myeloid dendritic cells ex vivo in patients with advanced cirrhosis [9]. However, it is not clear whether the imbalance of amino acids other than BCAAs influence the immune responses in patients with advanced cirrhosis. A previous study showed that the concentration of plasma L- Cystine (L-Cys) is higher in patients with cirrhosis and shows a wide range of variation [10]. Increased levels of L-Glutamine (L-Gln) and decreasing levels of L-Glutamate (L-Glu) are seen in patients with advanced cirrhosis, because the L-Glu-L-Gln exchange regulates the high levels of toxic ammonia in such patients [11]. Furthermore, previous studies demonstrated that antigen-presenting cells take up L-Cys via the Na-independent anionic amino acid transport system Xc^−^(4F2hc+xCT) and secrete substantial amounts of L-Glu, influencing the immune-responses through this exchange [12], [13], [14]. This transporter is composed of two protein components, xCT and 4F2hc (CD98), and the transport activity is thought to be mediated by xCT [15], [16]. The aim of this study is to investigate the influence of the extracellular L-Cys/L-Glu imbalance observed in patients with advanced cirrhosis on the function of peripheral monocytes using a serum-free culture medium with the average concentration of plasma amino acids from patients with advanced cirrhosis [9], thereby approximating the actual environment of the living body.

## Materials and Methods

### Ethics Statement

Written informed consent was obtained from each individual and the study protocol was approved by the Ethics Committee of Tohoku University School of Medicine (2009-209, 2009-535).

### Monocyte count and isolation

In patients with cirrhosis, the monocyte and lymphocyte counts were measured by a Beckman Coulter LH 750 Analyzer (Fullerton, CA, USA). PBMCs were separated from the peripheral blood of healthy volunteers or patients with cirrhosis by centrifugation on a density gradient. The CD14-positive monocytes were isolated from PBMCs using magnetic microbeads (Miltenyi Biotec, Bergish Gladbach).

### The serum free culture media used in this study

A serum free culture medium with the average concentration of plasma amino acids from healthy volunteers (HCM), that from patients with advanced cirrhosis (ACM) and complete culture media (CCM) were described previously [9]. Other components except amino acids were identical among media. Various concentrations of L-Cys were added to L-Cys free ACM , and the final concentration was adjusted to 0–300 nmol/mL (Table S1). We cultured CD14+monocytes, THP-1, Jurkat and Molt-4 under the these media with stimulant and measured the amino acid concentrations of these media. The viability of monocytes and PBMCs was determined using Annexin V^FITC^, with dead cells identified by propidium iodide (PI) staining (Annexin V^−FITC^ Apoptosis Detection Kit, BioVision, Mountain View, CA), according to the manufacturer's instructions. We confirmed the viability of PBMCs cultured in ACM and ACM plus L-Cys to be equal to that of complete culture medium (CCM) and X-VIVO 10 (Cambrex Bio Science Walkersville, Inc. Walkersville, MD USA).

### Patients and Healthy volunteers

The concentrations of the plasma amino acids from fasting patients with chronic hepatitis (n = 17), and patients with cirrhosis (n = 130) were measured by high-performance liquid chromatography (HPLC) in the early morning. Briefly, sulfosalicylic acid was added to the plasma to a final concentration of 5%. The samples were then placed on ice for 15 minutes followed by centrifugation to remove precipitated proteins. The extracts were then analyzed for the amino acid content with a JLC-500/V (Japan Electron Optics Laboratories, Tokyo, Japan). Also, the patients with cirrhosis were classified according to the Child-Pugh classification. We defined as Child-Pugh grade B or C the patients with advanced cirrhosis. The estimated glomerular filtration rate (eGFR) was calculated using the new Japanese equation [17].

We selected nineteen patients with cirrhosis for in vitro or ex vivo studies (Table S2). All of these patients were inpatients. The MELD score [18] was calculated by an on-line worksheet available on the internet at www.mayoclinic.org/meld/mayomodel5.html.

### Monocyte proliferation assay

Monocytes were cultured at a density of 2.5×10^5^ cells/well in 96-well plates containing each media with 1,000 U/mL M-CSF (PEPROTECH EC, London, UK). One half the amount of culture fluid was exchanged every one day. Cells were maintained for 20 days and the proliferation rate of the cells was measured using Carboxyfluorescein Succinimidyl Ester (CFSE) staining; CellTrace CFSE Cell Proliferation Kit (Molecular Probes, Oregon). The staining methods followed the manufacturer's protocol.

### Cytokine analysis

PBMCs or monocytes were preincubated at a density of 2.5×10^5^ cells/well in 96-well flat-bottom plates (CORNING, NY) for 2 hours in each of the media, and 100 ng/mL LPS (Escherichia coli 026:B6 (SIGMA) were added. The supernatants were collected after 24 hours and immediately TNF-alpha, IFN-gamma, IL-10, GM-CSF were determined by specific cytokine ELISA kits (Bender MedSystems) according to the manufacturer's instructions.

### Surface marker analysis

Monocytes were harvested and labeled with FITC-, PE- and APC-labeled monoclonal antibodies (mAbs) (anti-human CD14, CD80, CD86, CD98, HLA-DR, or the relevant isotype controls: BD PharMingen, San Diego, CA), according to the manufacturer's instructions. On xCT expression, indirect staining was performed; primary antibody (xCT (H-121) scc-98552: Santa Cruz) secondary antibody (goat anti-rabbit IgG-FITC sc-2012: Santa Cruz) Using a FACS Canto II (BD Immunocytometry Systems, San Diego, CA) flow cytometer, surface marker expressions were analyzed using the BD FACSDiva (BD Immunocytometry Systems) program.

### Intra-extracellular amino acid quantification

The THP-1, Jurkat and Molt4 cells were pre-incubated for 2 hr in ACM, then 1.0×10^7^ cells were re-suspended with LPS (100 ng/mL) or IL-2 (1000 IU/mL) in 1 mL of ACM, L-Cys-free ACM or ACM with L-Cys. After 2 hr incubation, the supernatants were measured by HPLC for the extracellular amino acid quantification. The concentration of the intracellular amino acids was determined as described in ref [19]. Briefly, cells were washed two times by PBS and resuspended in 500 µL PBS sonicated with four 10-s pulses. Cell debris was removed by centrifugation, and sulfosalicylic acid was added to the supernatant to a final concentration of 2%. The samples were then placed on ice for 30 min followed by centrifugation to remove precipitated proteins. The extracts were then analyzed for amino acid content with an L-8500 amino acid analyzer (Hitachi Ltd., Tokyo).

### Measurement of reduced glutathione (GSH) and oxidized glutathione (GSSG)

CD14+ monocytes were pre incubated at a density of 2.0×10^5^ cells/well in 96-well plates containing HCM for 2 h and then cultured in HCM, ACM or ACM plus L-Cys for an additional 2 h. The culture medium was carefully removed from the wells. 100 µl of prepared GSH-Glo™ Reagent were added to each well of a 96-well plate, mixed briefly on a plate shaker, and incubated at room temperature for 30 minutes. 100 µl of reconstituted Luciferin Detection Reagent were added to each well of a 96-well plate, mixed briefly on a plate shaker, and incubated for 15 minutes. luminescence was read by a Lumino Skan Ascent (Thermo BioAnalysis, Helsinki, Finland).

### Real-time PCR

THP-1, Jurkat, Molt-4 and CD14+ monocytes were collected. After the extraction of total RNA and the RT procedure, real-time PCR using aTaqMan Chemistry System () was carried out. The ready-made sets of primers and probes for the amplification of xCT (Assay ID : Hs00921937_m1), TNF-alpha (Assay ID : Hs99999043_m1) and glyceraldehyde-3-phosphate-dehydrogenase (GAPDH, Assay ID : Hs02758991_g1) were purchased from Perkin-Elmer/Applied Biosystems. The relative amount of target mRNA was obtained by using a comparative threshold cycle (CT) method. The expression level of mRNAs of the Molt-4 was represented as 1.0 and the relative amounts of target mRNA in THP-1 and Jurkat were calculated according to the manufacturer's protocol. For CD14+ monocytes, The expression level of monocyte mRNA from a healthy volunteer was represented as 1.0 and the relative amounts of target mRNA in monocytes from patients were calculated.

### Statistical Analysis

The data were analyzed with ANOVA, and multiple comparisons were performed with Dunnett's post-hoc procedure for the plasma aminogram. When 2 groups were analyzed, the differences between media were analyzed by the Wilcoxon t test, and the differences between healthy controls and patients were analyzed by the Mann-Whitney U-test. All statistical analyses were performed with standard statistical software (SPSS 13.0 for Windows, Chicago, IL).

## Results

### The counts of peripheral monocytes were increased in association with the plasma L-Cystine in patients with advanced cirrhosis

**Figure 1:**
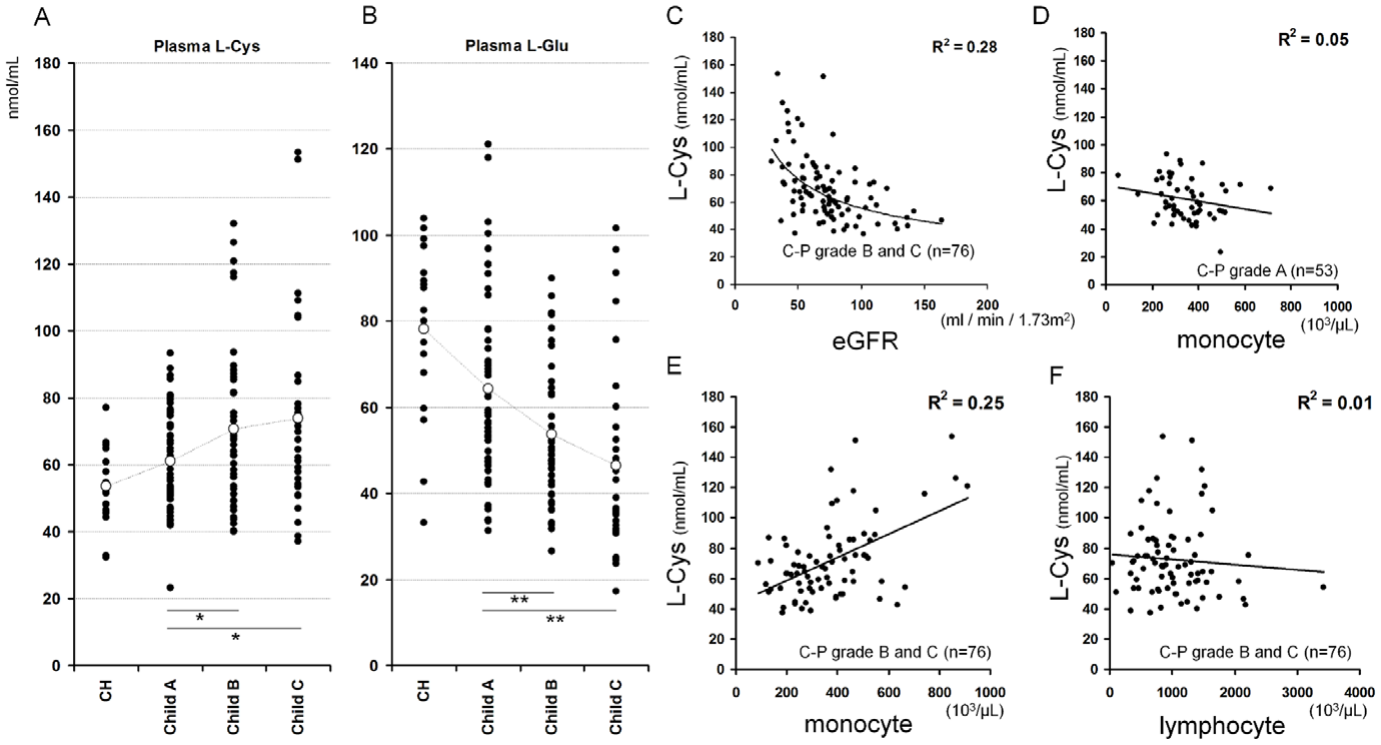
The counts of peripheral monocyte were increased in association with plasma L-Cystine in patients with advanced cirrhosis. A, B, The concentrations of plasma L-Cys in patients with cirrhosis was increased and that of plasma L-Glu was decreased acccording to Child-Pugh grade. The levels of plasma L-Cys and L-Glu in patients with cirrrhosis (n = 130) were measured using HPLC and classfied by the Child-Pugh classfication. C, Nonliner regression model was used to model variation in plasma L-Cys and eGFR. D, E,F, Linear regression model was used to model variation in plasma L-Cys and monocyte and lymphocyte counts. Individual correlations between plasma L-Cys levels and monocyte counts in patients with early cirrhosis (D), that in patients with advanced cirrhosis (E), and lymphocyte counts in patients with advanced cirrhosis (F). A,B, **, p<0.01, _*, p<0.05 vs Child-Pugh grade A. Statistical significance was determined by one-way ANOVA and Dunnett's post-hoc procedure. C, D, E, F, R^2^ represents coefficient of determination.

Firstly, we confirmed that, in patients with advanced cirrhosis (Child-Pugh grade B or C), the plasma concentrations of L-Cys were significantly higher than in those with early cirrhosis (Figure 1A), and there was a wide range of variation. On the other hand, the plasma concentrations of L-Glu were significantly decreased along with the Child-Pugh grade (Figure 1B). In patients with advanced cirrhosis, the wide range of variation of L-Cys was attributed to the eGFR (R^2^ = 0.28/P = 0.0000008) (Figure 1C). These data mean that plasma L-Cys increases in decompensated cirrhosis. Secondly, we investigate whether the concentration of plasma L-Cys influenced the peripheral monocyte counts. In patients with early cirrhosis, L-Cys was not correlated with the monocyte counts (R^2^ = 0.05/P = 0.119) (Figure 1D), but in patients with advanced cirrhosis, it was significantly and positively correlated with the monocyte counts (R^2^ = 0.25/P = 0.0000017) (Figure 1E). On the other hand, the lymphocyte counts were not correlated with the concentration of plasma L-Cys (R^2^ = 0.01/P = 0.523) (Figure 1F). Interestingly, among all twenty kinds of free amino acids, only L-Cys was significantly correlated with the monocyte counts in patients with advanced cirrhosis (Figure S1). These data mean that, in patients with advanced cirrhosis, plasma L-Cys is increased according to renal dysfunction and influences the counts of monocytes.

Extracellular L-Cystine dose-dependently increased pro-inflammatory cytokines from CD14+ monocytes under the amino acid condition of advanced cirrhosis

**Figure 2:**
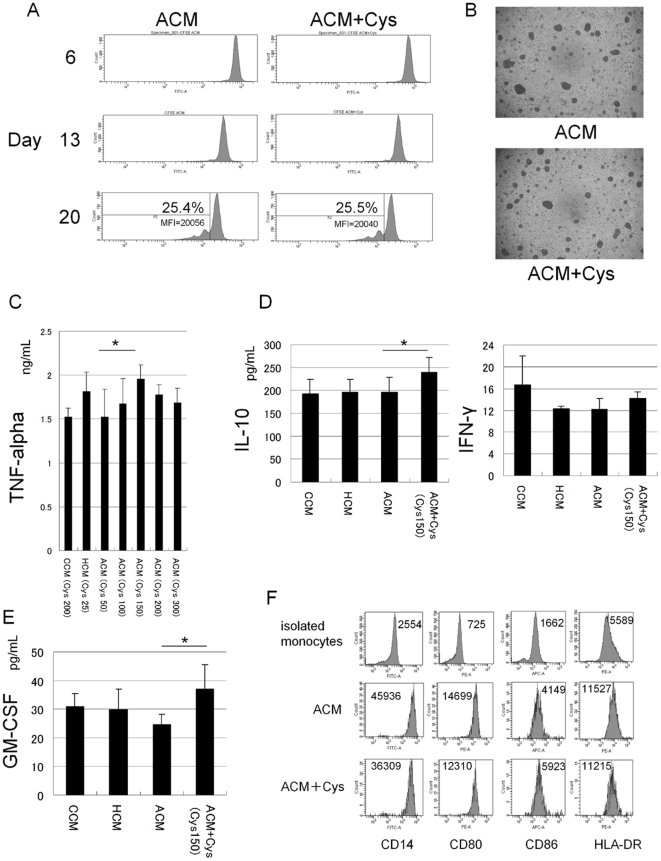
L-Cystine dose-dependently increased TNF-alpha from CD14+ monocytes with LPS under the amino acid environment of patients with advanced cirrhosis. A, Isolated CD14+ monocytes (purity >90%) were cultured at a density of 2.5×10^5^ cells/well in 96-well plates containing in ACM and ACM plus L-Cys (L-Cys : 150 nmol/mL) with 1,000 U/mL M-CSF. One half the amount of culture fluid was exchanged every one day. Cells were maintained for 20 days and the proliferation rate of the cells was measured using CFSE staining. B, Influence of L-Cys on microscopic appearance of monocyte proliferation under serum-free conditions. Day 20, cells in firmly adherent clusters in both ACM and ACM+Cys. C, Monocytes were cultured under CCM, HCM, ACM and ACM plus L-Cys (100–300 nmol/mL). Cells were pre-incubated at a density of 2.5×10^5^ cells/well in 96-well flat-bottom plates for 2 hours in each of the media, and 100 ng/mL LPS was added. The supernatants were collected after 24 hours and immediately TNF-alpha was determined by specific cytokine ELISA kits. D,E, Similarly as in Fig. 2C, IL-10, IFN gamma from monocytes and GM-CSF from PBMCs under CCM, HCM, ACM, ACM+Cys (L-Cys 150 nmol/mL) were measured with ELISA. F, Cells were harvested after 24 hours, stained with different mAbs, and analyzed using flow cytometry. Cells were stained with FITC-labeled anti-CD14, -CD80, -CD86, and -HLA-DR. A, B and F results are representative of four experiments from three different donors. C, D and E, Mean ± SEM values from five different donors are shown. C,D,E *, p<0.05 vs ACM (paired Student's t test, two-tailed).

Based on the result that the peripheral monocyte counts were positively correlated with the concentration of L-Cys, we hypothesized that the concentration of extracellular L-Cys could influence the proliferation of monocytes. To investigate this hypothesis, we cultured monocytes for 20 days with M-CSF under ACM or ACM plus L-Cys in vitro, and determined the proliferation of monocytes by CFSE assay. An elevated concentration of L-Cys did not influence the proliferation of monocytes (Figure 2A) on microscopic appearance, and also did not affect the morphological appearance and behavior of the cells in culture (Figure 2B). There was also no difference in the proliferation of the monocyte cell line, THP-1 between these media (data not shown). Next, to investigate whether the extracellular L-Cys level influenced the production of inflammatory cytokines from monocytes, we cultured monocytes under ACM that contained 50–300 nmol/mL L-Cys and measured the producton of TNF alpha from monocytes. The addition of L-Cys increased the production of TNF alpha from monocytes in a dose-dependent manner (Figure 2C), and the values were maximum under 150 nmol/mL L-Cys. Interestingly, this range was in remarkable agreement with the range in patients with advanced cirrhosis (Fig. 1A). The IL-10 level from monocytes was also significantly higher under ACM plus L-Cys than that under ACM (Figure 2D), and there was no difference the interferon gamma (IFN γ) level from monocytes between these media. The GM-CSF from PBMCs was also significantly higher under ACM plus L-Cys than that under ACM (Figure 2E). Regarding monocyte phenotypes, there was no difference between ACM and ACM plus L-Cys (Figure 2F). These data mean that high levels of extracellular L-Cys increased pro-inflammatory cytokines from CD14+ monocytes under the amino acid environment of patients with advanced cirrhosis.

### High levels of extracellular L-Cystine promoted the L-Cystine-L-Glutamate antiport and decreased the intracellular GSH/GSSG ratio in monocyte under the amino acid environment of patients with advanced cirrhosis

We investigated whether high levels of extracellular L-Cys influence the L-Cys/L-Glu transport under the amino acid environment of patients with advanced cirrhosis.

**Figure 3:**
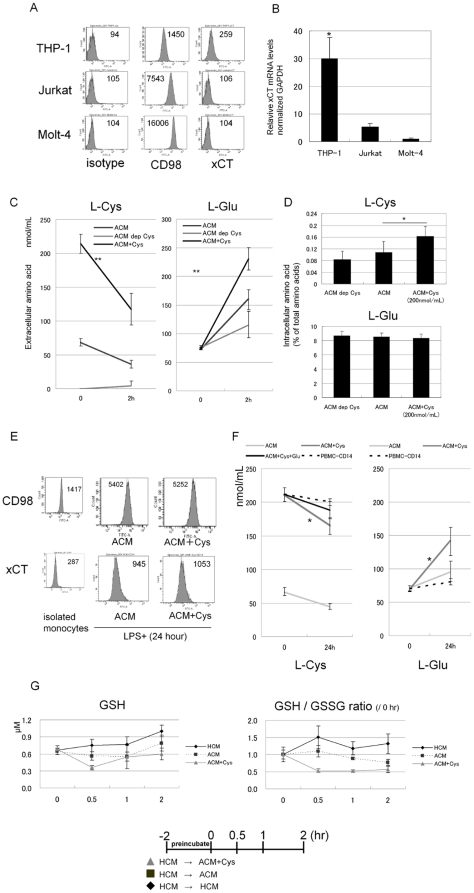
High levels of extracellular L-Cystine promoted the L-Cystine-L-Glutamate antiport via xCT and decreased the intracellular GSH/GSSG ratio in monocyte under the amino acid environment of patients with advanced cirrhosis. A, THP-1, Jurkat and Molt-4 cultured under CCM were harvested and labeled with antibodies (CD98, xCT or the relevant isotype controls). Using flow cytometry, surface marker expressions were analyzed. The figure expresses the mean fluorescence intensity. Data shown are representative of three independent experiments with cells. B, xCT relative mRNA levels of these cell lines were determined by real time PCR: delta-delta CT method. All mRNA expression levels were normalized to GAPDH. C, D, The THP-1 cells were pre-incubated for 2 hours in ACM, then resusupended with LPS (100 ng/mL) in 1 mL of ACM, L-Cys-free ACM and ACM plus L-Cys. The concentration of extracellular (C) and intracellular (D) amino acid was determined as described in material and methods. E, The CD14+ monocytes, cultured under ACM and ACM plus L-Cys for 24 hours, were harvested and labeled with antibodies (CD98, xCT or the relevant isotype controls). Using flow cytometry, surface marker expressions were analyzed. The figure expresses the mean fluorescence Intensity. Data shown are representative of three independent experiments with cells. F, Similarly as in Fig. 4C, the monocytes were cultured for 24 hours in ACM, L-Cys-free ACM and ACM plus L-Cys. The supernatants were measured by HPLC as extracellular amino acids quantification. G, Monocytes were pre incubated at a density of 2.0×10^5^ cells/well in 96-well flat-bottom plates for 2 hours in HCM, and then cultured in HCM, ACM and ACM plus L-Cys (200 nmol/mL) for an additional 2 hours. These intracellular glutathione levels were measured by GSH-Glo™ at the time point indicated. C and D, Mean ± SD values from five independent experiments are shown. B *, p<0.05 vs Molt-4 (the Mann-Whitney U-test.). C,F **, p<0.01 *,P<0.05 (mean change vs ACM) D *, p<0.05 (paired Student's t test, two-tailed).

Firstly, we determined the expression of 4F2hc (CD98) and xCT in THP-1, Jurkat and Molt-4. All cell lines expressed CD98, but only THP-1 expressed xCT at the protein level (Fig. 3A) and mRNA level (Fig. 3B). Secondly, we measured the intra-extracellular L-Cys and L-Glu concentration of THP-1 under ACM at various L-Cys levels. After 2 hours culture, ACM plus L-Cys significantly decreased the extracellular L-Cys (mean change, ACM+Cys, −96.8±15.8; ACM, −32.3±0.6 and ACM dep L-Cys, 4.8±6.6 nmol/mL) (Fig. 3C) and significantly increased intracellular L-Cys (Fig. 3D) and extracellular L-Glu (mean change, ACM+Cys, 157.5±19; ACM, 86.1±17.7 and ACM dep L-Cys, 40.4±24.9 nmol/mL) (Fig. 3C) more than that by ACM or ACM deprived of L-Cys. For intracellular L-Glu, there was no difference among these media. Such L-Cys/L-Glu changes were not seen for Jurkat and Molt-4 (data not shown). These data indicate that high levels of extracellular L-Cys enhances L-Cys/L-Glu antiport in the monocyte cell line THP-1. Similarly, CD14+ monocytes expressed CD98 and xCT after adding LPS (Fig. 3E) and extracellular L-Cys/L-Glu changes were seen (L-Cys mean change, ACM+Cys, −46.9±5.0; ACM, −22.4±6.0; ACM+Cys+Glu, −22.9±6.4 and PBMC-CD14, −11.0±11.9 nmol/mL/L-Glu mean change; ACM+Cys, 70.4±24.8; ACM, 25.2±13.8 and PBMC-CD14, 10.7±7.0 nmol/mL) (Fig. 3F). Furthermore, we investigated whether high levels of extracellular L-Cys influence the intracellular glutathione level of monocytes. Interestingly, the intracellular GSH and GSH/GSSG ratio decreased more under ACM plus L-Cys than under ACM or HCM (Fig. 3G).

### Plasma L-Cys/L-Glu ratio significantly correlated with plasma TNF-alpha level in patients with advanced cirrhosis

**Figure 4:**
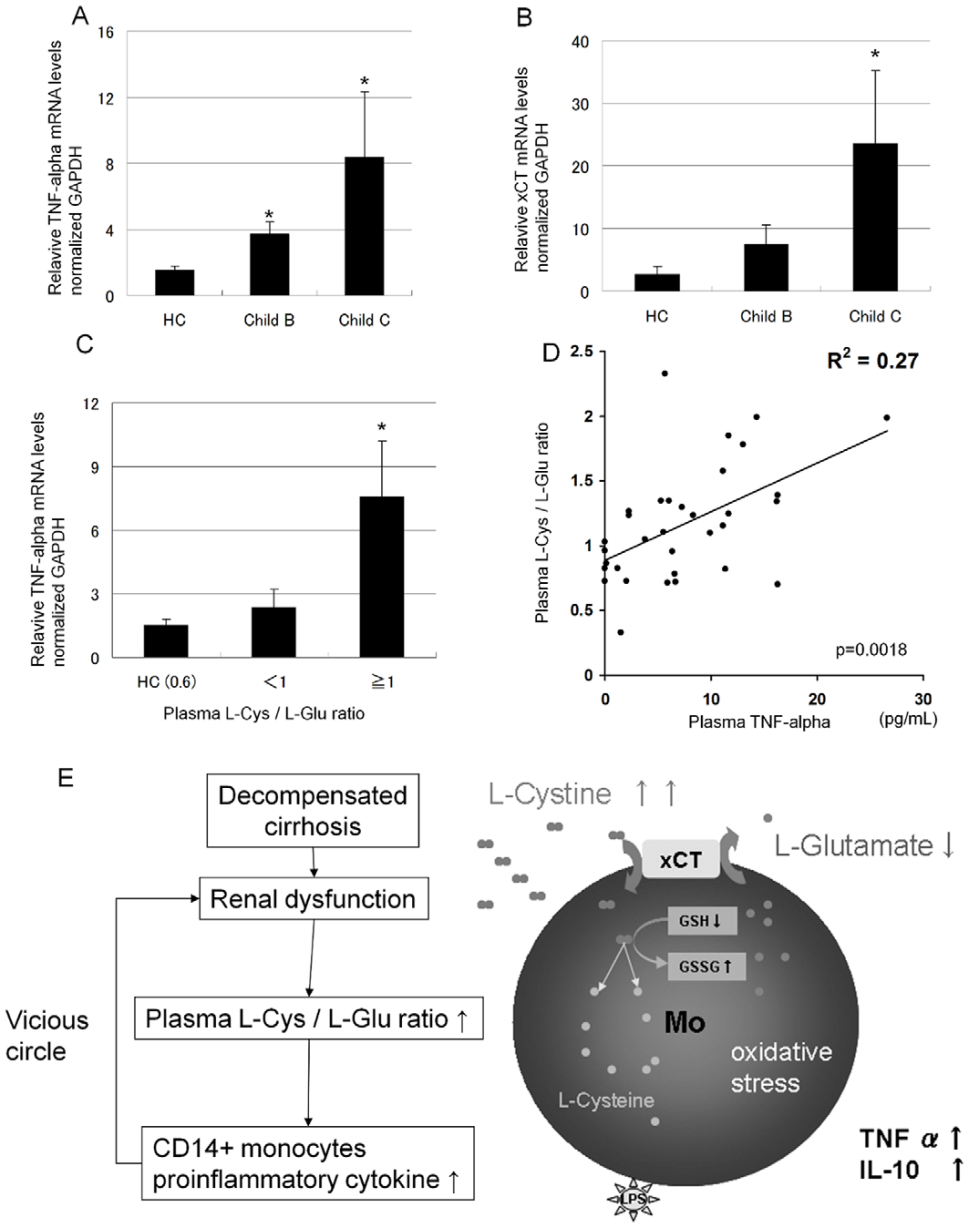
The plasma L-Cys/L-Glu ratio significantly correlated with the plasma TNF-alpha level in patients with advanced cirrhosis. A, CD14+monocytes were isolated from healthy volunteers (n = 5) and patients with adcanced cirrhosis (Table S2 : Patient 1–19). the TNF-alpha relative mRNA levels of CD14+monocytes were determined by real time PCR : delta-delta CT method. All mRNA expression levels were normalized to GAPDH. B, Similarly as in Fig. 4A, the xCT relative mRNA levels of monocytes were determined by real time PCR. C, These patients were separated into a high L-Cys/L-Glu ratio group (≥1) and low ratio group (<1). In healthy control (HC), the average plasma L-Cys/L-Glu ratio was 0.61±0.21. D, Linear regression model was used to model variation in plasma L-Cys/L-Glu ratio and plasma TNF-alpha. R^2^ represents coefficient of determination. E, The schematic diagram of the present study concerning monocytes abnormality in patients with decompensated cirrhosis. A,B,C *, p<0.05 vs HC (the Mann-Whitney U-test.).

Finally, we actually measured the levels of TNF-alpha of patients with advanced cirrhosis in monocytes and plasma (Table S2). In patients with advanced cirrhosis (Table S2: patients 1–19), the TNF-alpha mRNA expression of monocytes was significantly higher than that of healthy controls (Fig. 4A), and xCT mRNA expressions also was increased according to the Child-pugh grade (Fig. 4B). Interestingly, the TNF-alpha mRNA of monocytes was significantly higher in the high plasma L-Cys/L-Glu ratio group (≥1) than in the low group (<1) (Fig. 4C). Consistent with these data, the plasma TNF-alpha in the patients was significantly correlated with the plasma L-Cys/L-Glu ratio (p = 0.0018/r = 0.52247) (Fig. 4D). We represented the schematic diagram of the present study concerning monocytes abnormality in patients with decompensated cirrhosis (Fig. 4E).

## Discussion

Bacterial infections, such as spontaneous bacterial peritonitis (SBP) or pneumonia, are frequent clinical complications and causes of death in patients with advanced cirrhosis [20], because in such immune-compromised patients the innate immune cells can not normally respond to the pathogen [21]. Neutrophils, macrophages, and DCs are important cellular mediators of the innate immune defense. Circulating monocytes, however, are increasingly implicated as essential players in the defense against a range of microbial pathogens [22]. Previously, we made two serum-free media (HCM and ACM) to examine more closely the actual amino acid environment of the living body plasma [9]. First, we showed that plasma L-Cys was increased by renal dysfunction, which is an important factor of the MELD score [18], and showed a significantly positive correlation with the monocyte counts in patient with advanced cirrhosis. However, high levels of L-Cys did not directly influence the proliferation of monocytes in vitro. This paradox raises the possibility that the GM-CSF from PBMCs (Fig. 2E) may indirectly increase the peripheral monocyte counts, because the increase is almost entirely due to the release from bone marrow [23]. This issue should be evaluated in future studies. Second, we showed that extracellular L-Cys dose-dependently increases pro-inflammatory cytokines from monocytes with LPS under the amino acid environment of patients with advanced cirrhosis. Concerning the mechanism that underlies these phenomena, we confirmed that high extracellular levels of L-Cys enhanced the exchange L-Cys/L-Glu antiport of monocytes via xCT, and decreased the intracellular GSH/GSSG ratio under the amino acid condition of advanced cirrhosis. A previous study showed that oxidized Eh L-Cysteine/L-Cys induces the upregulation of nuclear factor-kappa B of monocytes in vitro [24]. These studies support our results. However, the same studies reported that the oxidized extracellular Cys/CySS redox state had no effect on cellular GSH/GSSG redox [24], [25]. We think that such differences were probably caused by differences in the culture condition and stimulation period of the immune cells, and that our culture conditions more closely matched the actual amino acid environment of patients with advanced cirrhosis. However, we need to investigate in detail by separate quantification of the reduced form, L-Cysteine; the oxidized form, L-Cys; and the mixed protein L-Cysteine disulfide. Furthermore, we think that a low level of plasma L-Glu enhances the antiport in patients with advanced cirrhosis, because another study reported on a L-Cys transport system whose activity was inhibited by L-Glu in mammalian cultured cells [26].

Finally, we confirmed that the TNF-alpha mRNA of CD14 monocytes, isolated from patients with advanced cirrhosis, was at a higher level than in healthy controls. Furthermore, the value of plasma TNF-alpha showed a significantly positive correlating with the plasma L-Cys/L-Glu ratio.

This present results still cannot be construed as conclusive evidence of a change in the immune system in patients with advanced cirrhosis. We need to investigate whether L-Cys/L-Glu imbalance influences other immune cells such as macrophages, dendritic cells, T-cells and B-cells, and their interaction, and whether the level of L-Glu influences the immune system, because previous studies reported that glutamate is a immunomediator in the intercellular cross-talk between DC and T cells [12], [14], [27]. In conclusion, we demonstrated for the first time that an L-Cys/L-Glu imbalance, especially high levels of L-Cys, increases pro-inflammatory cytokines, especially TNF-alpha from peripheral CD14+ monocytes under the amino acid condition of advanced cirrhosis in vitro, and these results are consistent with the relationships among plasma L-Cys and TNF-alpha in patients with advanced cirrhosis. This study may provide a new approach for future studies to ameliorate the immune dysfunction in patients with advanced cirrhosis.

## Supporting Information

Figure S1 Linear regression model was used to model variation in plasma L-Cys and monocyte count. Among all twenty kinds of free amino acids, only L-Cys was significantly correlated with the monocyte counts in patients with advanced cirrhosis.
(TIF)

Table S1 The serum free culture media used in this study. ‘ACM (advanced cirrhotic medium) consistent with the average concentration of plasma amino acids from patients (Child-Pugh grade B or C, n = 90). ACM+Cys: Varying concentrations of L-Cys were added to L-Cys-free ACM, and the final concentration was adjusted to 100–300 nmol/mL. ACM dep Cys: L-Cys free ACM. Other components except amino acids, were identical among media. The amino acid concentrations are expressed in nmol/mL. Fischer's ratio = (Valine+Leucine+Isoleucine)/(Tyrosine+Phenylalanine). We verified that there was no difference between the theoretical value and actual value examined by high performance liquid chromatography.
(DOC)

Table S2 Characteristics of study participants. LC-C: liver cirrhosis due to HCV LC-B: liver cirrhosis due to HBV HCC: hepatocellular carcinoma PBC: Primary biliary cirrhosis Alcoholic: Alcoholic cirrhosis NASH: non alcoholic steatohepatitis HA: Hepatic Encephalopathy PLT: platelet counts (×10^3^/µL) PT-INR: prothrombin time-international normalized ratio AST/ALT: aspartate amino transferase/alanine amino transferase (IU/L) Total Bilirubin (mg/dL) Albumin (g/dL) Fischer's ratio mean: L-Valine+L-Leucine+L-Isoleucine/L-Tyrosine+L-Phenylalanine.
(DOC)
