# Supplementary figures and images for: Plasma L-Cystine/L-Glutamate Imbalance Increases Tumor Necrosis Factor-Alpha from CD14+ Circulating Monocytes in Patients with Advanced Cirrhosis
Source: PLoS One. 2011 Aug 17;6(8):e23402. doi: 10.1371/journal.pone.0023402 (PMC3157377; doi:10.1371/journal.pone.0023402)

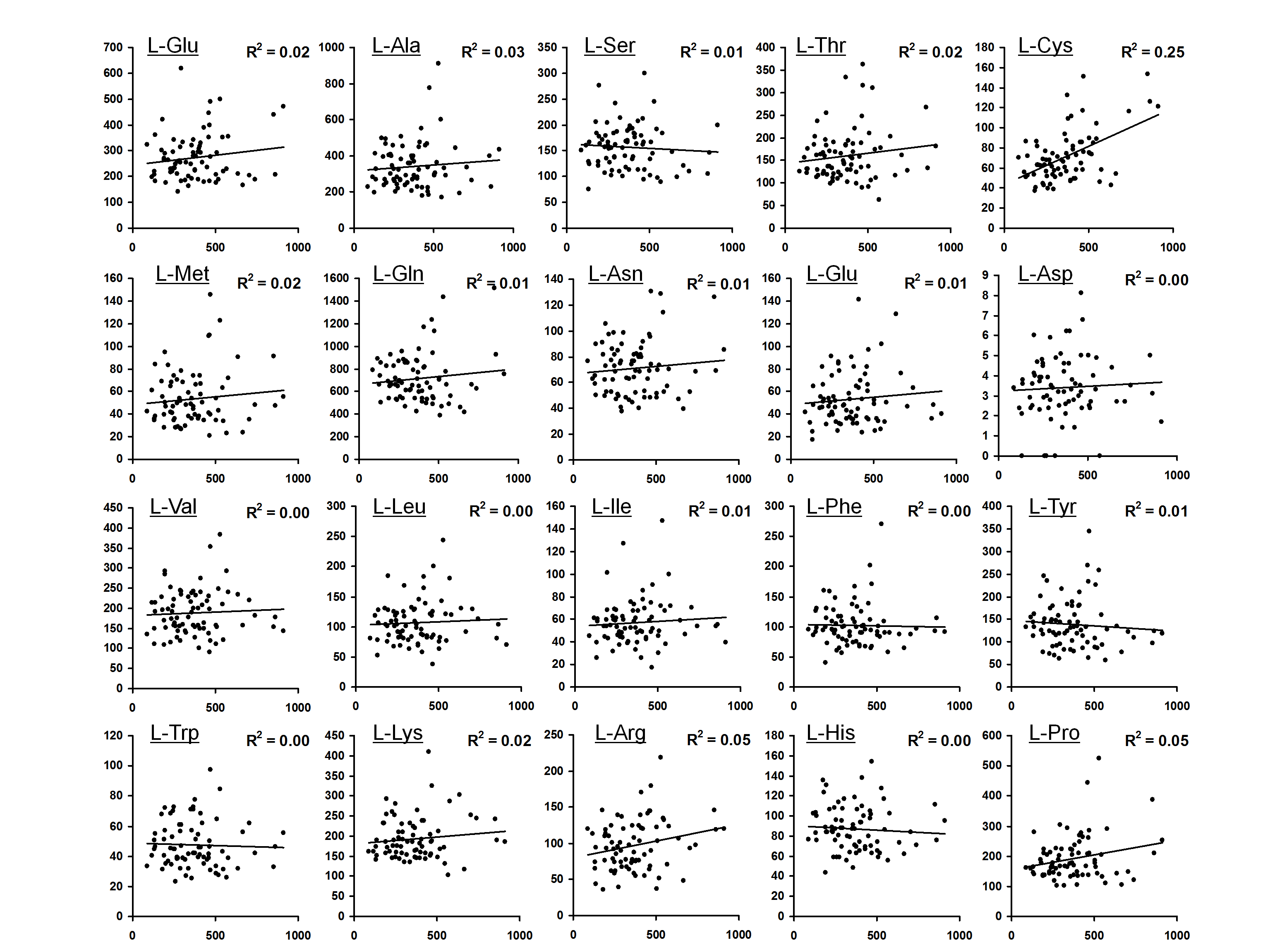

Supplement: Figure S1 — Linear regression model was used to model variation in plasma L-Cys and monocyte count. Among all twenty kinds of free amino acids, only L-Cys was significantly correlated with the monocyte counts in patients with advanced cirrhosis. (TIF) [file pone.0023402.s001.tif]
