# Supplementary material for: Plasma L-Cystine/L-Glutamate Imbalance Increases Tumor Necrosis Factor-Alpha from CD14+ Circulating Monocytes in Patients with Advanced Cirrhosis
Source: PLoS One. 2011 Aug 17;6(8):e23402. doi: 10.1371/journal.pone.0023402 (PMC3157377; doi:10.1371/journal.pone.0023402)
Supplement: Table S1 — The serum free culture media used in this study. ‘ACM (advanced cirrhotic medium) consistent with the average concentration of plasma amino acids from patients (Child-Pugh grade B or C, n = 90). ACM+Cys: Varying concentrations of L-Cys were added to L-Cys-free ACM, and the final concentration was adjusted to 100–300 nmol/mL. ACM dep Cys: L-Cys free ACM. Other components except amino acids, were identical among media. The amino acid concentrations are expressed in nmol/mL. Fischer's ratio = (Valine+Leucine+Isoleucine)/(Tyrosine+Phenylalanine). We verified that there was no difference between the theoretical value and actual value examined by high performance liquid chromatography. (DOC) [file pone.0023402.s002.doc]

Table S1 The serum free culture media used in this study (nmol/mL).

|  | ACM | ACM+Cys | ACM dep Cys |
| --- | --- | --- | --- |
| Glycine | 280 | 280 | 280 |
| L-Alanine | 307 | 307 | 307 |
| L-Serine | 151 | 151 | 151 |
| L-Threonine | 138 | 138 | 138 |
| L-Cystine 2HCl | 67 | 100-300 | 0 |
| L-Methionine | 75 | 75 | 75 |
| L-Glutamine | 689 | 689 | 689 |
| L-Asparagine | 64 | 64 | 64 |
| L-Glutamic Acid | 53 | 53 | 53 |
| L-Aspartic Acid | 4 | 4 | 4 |
| L-Valine | 175 | 175 | 175 |
| L-Leucine | 100 | 100 | 100 |
| L-Isoleucine | 53 | 53 | 53 |
| L-Phenylalanine | 99 | 99 | 99 |
| L-Tyrosine | 133 | 133 | 133 |
| L-Tryptophan | 45 | 45 | 45 |
| L-Lysine-HCl | 184 | 184 | 184 |
| L-Arginine-HCl | 92 | 92 | 92 |
| L-Histidine HCl-H2O | 85 | 85 | 85 |
| L-Proline | 176 | 176 | 176 |
| Fisher's rate | 1.42 | 1.42 | 1.42 |
