# Supplementary material for: Plasma L-Cystine/L-Glutamate Imbalance Increases Tumor Necrosis Factor-Alpha from CD14+ Circulating Monocytes in Patients with Advanced Cirrhosis
Source: PLoS One. 2011 Aug 17;6(8):e23402. doi: 10.1371/journal.pone.0023402 (PMC3157377; doi:10.1371/journal.pone.0023402)
Supplement: Table S2 — Characteristics of study participants. LC-C: liver cirrhosis due to HCV LC-B: liver cirrhosis due to HBV HCC: hepatocellular carcinoma PBC: Primary biliary cirrhosis Alcoholic: Alcoholic cirrhosis NASH: non alcoholic steatohepatitis HA: Hepatic Encephalopathy PLT: platelet counts (×103/µL) PT-INR: prothrombin time-international normalized ratio AST/ALT: aspartate amino transferase/alanine amino transferase (IU/L) Total Bilirubin (mg/dL) Albumin (g/dL) Fischer's ratio mean: L-Valine+L-Leucine+L-Isoleucine/L-Tyrosine+L-Phenylalanine. (DOC) [file pone.0023402.s003.doc]

Table S2 Characteristics of study participants.

| Patient Number | Disease | Gender | Age | AST | ALT | Total Bilirubin | Albumin | PT-INR | PLT | Ascites | HA | L-Cys | L-Glu | L-Cys / L-Glu ratio | MELD score | Child-Pugh Classification | Plasma Fischer's ratio |
| --- | --- | --- | --- | --- | --- | --- | --- | --- | --- | --- | --- | --- | --- | --- | --- | --- | --- |
| 1 | LC-C | M | 58 | 75 | 54 | 2.5 | 3.1 | 1.75 | 49 | + | - | 58.9 | 42.4 | 1.39 | 13 | C (10) | 1.00 |
| 2 | LC-B | M | 52 | 41 | 28 | 3.2 | 2.6 | 1.41 | 77 | + | + | 71.1 | 30.6 | 2.32 | 15 | C (10) | 0.85 |
| 3 | LC-C/HCC | M | 65 | 48 | 26 | 2.3 | 2.3 | 1.06 | 82 | - | - | 69.8 | 44.3 | 1.58 | 8 | B (8) | 1.70 |
| 4 | PSC | M | 72 | 129 | 104 | 28.2 | 2.2 | 2.5 | 99 | + | - | 72.8 | 58.4 | 1.25 | 30 | C (12) | 1.14 |
| 5 | LC-C/HCC | M | 82 | 436 | 161 | 10.6 | 2.5 | 1.62 | 252 | + | - | 140.6 | 171.5 | 0.82 | 30 | C (11) | 1.09 |
| 6 | LC-B/HCC | M | 52 | 90 | 42 | 2.6 | 2.8 | 1.45 | 116 | + | - | 61.4 | 45.8 | 1.34 | 13 | B (9) | 1.26 |
| 7 | PBC | F | 59 | 103 | 37 | 4.9 | 2.5 | 1.17 | 134 | + | - | 84.3 | 45.6 | 1.85 | 13 | B (9) | 2.23 |
| 8 | LC-B/HCC | M | 57 | 203 | 150 | 17.7 | 2.5 | 1.52 | 19 | + | - | 92.4 | 92.4 | 1.00 | 20 | C (11) | 1.15 |
| 9 | LC-C/HCC | M | 73 | 85 | 60 | 0.7 | 3.1 | 1.01 | 103 | + | - | 22.9 | 41.9 | 0.55 | 5 | B (7) | 1.54 |
| 10 | LC-C/HCC | F | 78 | 39 | 18 | 0.9 | 2.4 | 1.23 | 59 | - | - | 65 | 28.3 | 2.30 | 7 | B (7) | 1.47 |
| 11 | LC-C/HCC | M | 68 | 65 | 43 | 0.6 | 2.1 | 1.11 | 145 | - | - | 71.2 | 74.2 | 0.96 | 4 | B (7) | 2.56 |
| 12 | LC-B/HCC | M | 56 | 57 | 68 | 8.9 | 2.6 | 1.05 | 106 | + | - | 67.4 | 104.4 | 0.65 | 15 | C (11) | 1.54 |
| 13 | LC-C/HCC | F | 70 | 51 | 48 | 2.8 | 2.0 | 1.55 | 83 | + | - | 71.8 | 32.9 | 2.18 | 10 | B (9) | 0.86 |
| 14 | LC-C/HCC | M | 60 | 78 | 47 | 2.3 | 2.5 | 1.24 | 46 | - | - | 57.1 | 30.8 | 1.85 | 10 | B (8) | 1.23 |
| 15 | LC-B | M | 66 | 111 | 106 | 4.5 | 3.1 | 1.67 | 8.9 | + | - | 36.1 | 48 | 0.75 | 18 | B (9) | 1.82 |
| 16 | LC-C/HCC | M | 79 | 79 | 44 | 1.5 | 2.2 | 1.25 | 14.2 | + | - | 72.9 | 24.6 | 2.96 | 10 | B (9) | 1.23 |
| 17 | LC-C/HCC | M | 67 | 104 | 57 | 1.0 | 2.6 | 1.27 | 120 | + | - | 30.8 | 36.7 | 0.84 | 9 | B (8) | 2.55 |
| 18 | LC-C/HCC | F | 74 | 59 | 30 | 2.1 | 2.4 | 1.47 | 30 | - | - | 47.9 | 38 | 1.26 | 9 | B (8) | 1.20 |
| 19 | PBC | F | 29 | 441 | 212 | 38.9 | 2.5 | 1.9 | 58 | + | - | 51 | 27 | 1.89 | 25 | C (13) | 2.09 |
| 20 | Alcoholic | M | 54 | 53 | 24 | 2.5 | 3.1 | 1.6 | 219 | + | + | 104.1 | 52.5 | 1.98 | 18 | C (10) | 1.24 |
| 21 | HBV | M | 60 | 58 | 28 | 4.7 | 2.1 | 1.8 | 48 | + | - | 62.1 | 31.2 | 1.99 | 15 | C (11) | 0.92 |
| 22 | cryptogenic | M | 75 | 65 | 54 | 1.0 | 3.5 | 1.04 | 206 | + | + | 117.4 | 65.9 | 1.78 | 9 | B (8) | 2.98 |
| 23 | Alcoholic | F | 44 | 33 | 16 | 6.5 | 2.3 | 1.87 | 95 | - | - | 57.9 | 50.1 | 1.16 | 20 | C (10) | 1.98 |
| 24 | LC-C/HCC | M | 47 | 76 | 47 | 5.7 | 2.7 | 1.9 | 38 | - | + | 42.8 | 38.9 | 1.10 | 14 | C (11) | 1.24 |
| 25 | LC-C/HCC | M | 76 | 83 | 75 | 1.4 | 2.5 | 1.17 | 103 | - | - | 81.4 | 66 | 1.23 | 6 | B (7) | 1.87 |
| 26 | PBC | F | 73 | 38 | 27 | 1.3 | 2.6 | 1.35 | 69 | + | + | 51.3 | 39.6 | 1.30 | 6 | B (8) | 1.37 |
| 27 | LC-C/HCC | F | 81 | 54 | 47 | 0.9 | 2.8 | 1.28 | 118 | + | - | 39.9 | 55.6 | 0.72 | 2 | B (7) | 2.19 |
| 28 | HBV | M | 58 | 76 | 27 | 6.0 | 2.9 | 2.22 | 51 | + | + | 75.5 | 96.7 | 0.78 | 20 | C (12) | 1.25 |
| 29 | LC-C/HCC | M | 68 | 156 | 134 | 1.9 | 3.3 | 1.15 | 65 | - | - | 74.6 | 78 | 0.96 | 7 | A (6) | 1.95 |
| 30 | LC-C/HCC | M | 78 | 31 | 20 | 1.6 | 2.7 | 1.59 | 93 | + | - | 56.4 | 41.9 | 1.35 | 10 | B (7) | 0.98 |
| 31 | PSC | M | 57 | 124 | 59 | 7.7 | 2.9 | 1.27 | 428 | - | - | 54.1 | 75.8 | 0.71 | 13 | B (8) | 2.09 |
| 32 | Alcoholic/HCC | M | 81 | 39 | 29 | 1.2 | 3.1 | 1.17 | 109 | - | - | 68.9 | 62.5 | 1.10 | 4 | A (6) | 1.71 |
| 33 | LC-B/HCC | M | 78 | 45 | 26 | 1.2 | 2.8 | 1.34 | 84 | - | - | 56.4 | 41.9 | 1.35 | 8 | B (7) | 0.98 |
| 34 | LC-C/HCC | F | 79 | 72 | 59 | 1.0 | 2.9 | 1.06 | 76 | - | - | 61.6 | 58.6 | 1.05 | 4 | A (6) | 1.84 |
| 35 | LC-C/HCC | F | 76 | 52 | 32 | 1.0 | 2.3 | 1.15 | 67 | - | - | 63 | 49.9 | 1.26 | 6 | B (7) | 1.39 |
| 36 | NASH | F | 68 | 76 | 48 | 2.2 | 3.0 | 1.8 | 129 | - | + | 64 | 51.9 | 1.23 | 11 | B (9) | 1.01 |
| 37 | PBC | F | 62 | 120 | 52 | 4.3 | 2.2 | 1.64 | 116 | + | - | 47 | 64.9 | 0.72 | 6 | C (10) | 1.43 |
| 38 | LC-C/HCC | M | 59 | 170 | 74 | 2.3 | 2.8 | 1.13 | 73 | - | - | 42.5 | 128.4 | 0.33 | 8 | B (8) | 1.90 |
| 39 | LC-C/HCC | M | 66 | 109 | 113 | 1.1 | 3.4 | 1.13 | 79 | - | - | 56.1 | 68.2 | 0.82 | 6 | A (6) | 2.09 |
| 40 | LC-C/HCC | M | 65 | 107 | 65 | 1.4 | 3.2 | 1.21 | 103 | + | - | 70.4 | 81.9 | 0.86 | 9 | B (7) | 1.52 |
| 41 | PBC | F | 58 | 98 | 70 | 6.7 | 3.1 | 1.36 | 141 | + | - | 74.3 | 90.1 | 0.82 | 10 | B (9) | 1.05 |
| 42 | Alcoholic | F | 33 | 94 | 22 | 15.0 | 2.0 | 2.37 | 164 | + | + | 57.8 | 60.2 | 0.96 | 20 | C (12) | 0.45 |
| 43 | PBC | F | 62 | 83 | 30 | 2.3 | 2.5 | 1.11 | 207 | + | + | 104.5 | 101.7 | 1.03 | 13 | C (10) | 1.29 |
